# Practical Medical Management of Crohn's Disease

**DOI:** 10.1155/2013/208073

**Published:** 2013-11-07

**Authors:** Bulent Baran, Cetin Karaca

**Affiliations:** ^1^Van Research and Training Hospital, Van 65300, Turkey; ^2^Department of Gastroenterohepatology, Istanbul Faculty of Medicine, Istanbul University, Istanbul 34093, Turkey

## Abstract

Crohn's disease is a chronic inflammatory disease of diagnostic and therapeutic challenges. After proper diagnosis, treatment decisions must be made on precise clinical judgment. During the course of the disease there are variable clinical features, so each case must be managed individually. Physicians who care for patients with Crohn's disease should be prepared for treatment options in different states of the disease and possible complications of both the disease and medications. This paper will focus on the management of Crohn's disease. We aim to discuss current treatment options in different presentations of the disease and to provide algorithmic management strategy.

## 1. General Principles of Management

Crohn's disease can affect any area of the gastrointestinal tract. Transmural inflammation and segmental pattern are the classical features of the disease [1]. A treatment plan should be organized according to disease activity, behavior and localization of disease, and associated complications. Whatever treatment plan is chosen, it is most appropriate to individualize treatment according to clinical response and tolerance of the patient. It is certain that smoking is an independent risk factor for complications and has a direct influence on disease activity. All patients diagnosed with Crohn's disease should be informed about the negative impact of smoking and strongly encouraged to cease smoking [2].

Nonsteroidal anti-inflammatory drug usage is known to be associated with mucosal damage in gastrointestinal tract. There is substantial evidence that exacerbation of inflammatory bowel disease occurs after nonsteroidal anti-inflammatory drug usage, although the available data is conflicting to make definitive conclusions. Regarding the mechanisms of relapse, the inhibition of prostaglandin synthesis appears to be the hallmark of the nonsteroidal anti-inflammatory drug effects [3–5]. The patients should be informed about the adverse effects of nonsteroidal anti-inflammatory drugs, and limited usage must be ensured.

As a principle current treatment options are chosen sequentially from safer drugs with less adverse events to more potent and potentially more toxic drugs to induce clinical remission. In this “step-up” strategy, main purpose is to induce and maintain remission by safer and less expensive drugs by identifying patients who will benefit from conventional treatments. However, some authors recently suggested using a “top-down” strategy in which more potent treatment options are chosen early in the course of the disease, particularly in patients with severely active Crohn's disease and an increased risk of complications. Most of the evidence that supports the top-down immunosuppressive strategy for autoimmune diseases comes from studies in rheumatoid arthritis. Currently, results of the studies comparing the efficacy of these two strategies in Crohn's disease are not convincing enough to draw a conclusion. In a large controlled trial that compares both strategies by D'Haens and colleagues, 133 treatment naïve patients with Crohn's disease were randomized to combined immunosuppression (infliximab and azathioprine) or conventional treatment (corticosteroids followed by azathioprine and/or infliximab) arms [6]. The number of patients in clinical remission was significantly more in combined immunosuppression arm at 26th week (60% versus 36%), although at the end of the 1st and 2nd year the significance was lost. Also, after 2 years there were much more patients (73 versus 30 patients) with mucosal healing in the early combined immunosuppression arm without increased adverse events. In the recent SONIC trial by Colombel and colleagues, patients with moderate to severe Crohn's disease who were refractory to treatment with mesalamine and/or corticosteroids were randomized to receive azathioprine monotherapy, infliximab monotherapy, or infliximab and azathioprine combination therapy [7]. At the end of followup (50th week) infliximab mono- and combination therapy arms produced significantly higher corticosteroid-free remission rates than azathioprine monotherapy (35%, 46% versus 24%, resp.). Mucosal healing was achieved in 44%, 30% and 16.5% of patients in combination, infliximab and azathioprine monotherapy arms, respectively. The risk of serious infections was similar in all treatment arms. Despite these encouraging results, the top-down approach is not appropriate for all patients, as most of them will not develop complicated disease. Some patients may greatly benefit from conventional immunosuppressives which means nearly 30% of patients may be exposed to unnecessary immunosuppression with widespread application of the top-down approach. Introduction of biologic agents early in the disease course may increase the risk of malignancies and infections. The high costs of these drugs also prohibit this strategy as a universal approach. In routine practice, top-down strategy may be selected at baseline particularly in patients that are predicted to have a complicated disease course. The baseline features of a possible complicated disease course are young presentation, male sex, and presence of perianal disease, fistulizing and/or stenosing behavior, and early need for surgery [8]. Still there is a need for studies to evaluate the risks and benefits of top-down strategy.

Whatever strategy is chosen, the patients should be evaluated in one to two weeks after the start of the treatment and followedup periodically. Follow-up intervals are determined individually according to the chosen treatment and patient characteristics. It is expected to have improvement in the first 2–4 weeks and to gain maximal effect in 12–16 weeks of therapy. The remission induction therapy is switched to maintenance therapy after clinical remission is achieved; however, alternative strategies will be necessary in treatment failure [1].

## 2. Assessment of Disease Severity

An assessment of disease severity is necessary to design an appropriate treatment plan. A patient with a mildly to moderately active Crohn's disease may benefit from oral budesonide, but it is inappropriate in oral intolerance and severely active disease with systemic symptoms as fever in which parenteral corticosteroids should be necessary. In clinical trials Crohn's Disease Activity Index (CDAI) and Harvey-Bradshaw Index are widely used disease assessment scores, but in clinical practice more convenient methods are usually preferred [1, 9]. A simplified classification of severity that we use in clinical practice is summarized next (Table 1).

- Clinical remission (CDAI < 150): spontaneous or posttreatment remission.
- Mild to moderate Crohn's disease (CDAI 150–220): good oral intake, absence of dehydration, abdominal tenderness/mass, obstruction, or weight loss of >10%. Ambulatory followup is sufficient.
- Moderate to severe Crohn's disease (CDAI 220–450): patients with mild to moderate Crohn's disease irresponsive to first line therapy; presence of 2 or more of the following systemic symptoms: fever, weight loss, abdominal pain, nausea and vomiting, and anemia.
- Severe-fulminant Crohn's disease (CDAI > 450): ambulatory patients with persisting symptoms despite optimal therapy, presence of high fever, or obstruction symptoms as refractory nausea/vomiting, peritoneal signs, cachexia, or intraabdominal abscess.

## 3. Medical Treatment of Crohn's Disease

There are a number of alternatives in treatment of Crohn's disease (Table 2). General approach however is universal for most of the cases, but individual patients may need exceptional treatment strategies coordinated by a multidisciplinary team including a gastroenterologist, surgeon, and radiologist. In general, treatment plan is determined according to the disease severity, behavior, and location. Previous treatment failures should be kept in mind while deciding for a future strategy. Proper and timely definition of response is crucial to decide if the therapy is effective and to initiate alternative treatments without a delay in case of inadequate response. It is also important in a clinical trial setting to correctly describe different response levels while comparing different treatment arms. Response to therapy is classified as nonresponse, clinical response, clinical remission and endoscopic remission, or mucosal healing. Clinical response should be defined as reduction in CDAI ≥ 100 points according to European Crohn's and Colitis Organization (ECCO) guidelines, although it has been defined as reduction in CDAI ≥ 70 points in several studies [10]. Clinical remission describes an asymptomatic patient with a CDAI score less than 150 points. A clinically asymptomatic patient with a normal CRP level may not necessarily have complete mucosal healing. In recent years, mucosal healing or endoscopic remission has been found to be an ideal target to achieve through novel therapies. Growing evidence suggest that it is the best marker of sustained remission and good prognosis; nevertheless aiming clinical remission is more rational and attainable despite novel therapeutic options. 

## 4. Mild to Moderate Ileocolonic Crohn's Disease

In this group of patients budesonide 9 mg/day is preferred for the induction of clinical remission. It was shown that budesonide is significantly more effective for induction of remission compared to placebo and 5-aminosalicylates (mesalamine) 4 g/day, and remission is achieved in 51–60% patients in 8–10 weeks [11]. In mild disease budesonide should be preferred to systemic corticosteroids due to reduced incidence of glucocorticoid associated adverse events.

There is an uncertainty about the benefit of widely used 5-aminosalicylate preparations in mild ileal Crohn's disease. In a meta-analysis, 5-aminosalicylate 4 g/day was shown to have a little or no effect for induction of remission in active ileocaecal Crohn's disease when compared to placebo [12]. In a more recent meta-analysis in 2011, 22 randomized trials were included to examine the role of 5-aminosalicylates in patients with Crohn's disease for both induction of remission and maintenance of remission [13]. Failure to induce remission was seen in 68% of patients treated with 5-aminosalicylates compared with 75% of patients treated with placebo (relative risk 0.89; 95% CI 0.80–0.99). The number needed to treat was found to be 11 in active Crohn's disease. In summary, 5-aminosalicylates taken as a group were superior to placebo for the induction of remission, but they are inferior to glucocorticoids. Also, there was no benefit with 5-aminosalicylate treatment for the maintenance of remission. Patients treated with 5-aminosalicylate had a relapse rate of 56 percent compared with 57 percent for patients who received placebo. Despite controversy 5-aminosalicylates are used because of their relative safety compared to other drugs such as corticosteroids, immunomodulators, and biologic agents. Although current guidelines recommend against its use [2], some suggest that 5-aminosalicylates should be used in patients with Crohn's colitis particularly because of the potential chemopreventive benefits in patients with longstanding disease. In fact a recent population based study reported that 5-aminosalicylates are not chemoprophylactic for colorectal cancer in inflammatory bowel disease [14].

Different 5-aminosalicylate preparations include sulfasalazine and mesalamine formulations. Sulfasalazine is an azobonded compound which is mainly active in colon where it is reduced by the bacterial enzyme azoreductase to sulfapyridine and 5-aminosalicylate. Due to need for coliform bacteria to produce its active moieties, sulfasalazine is not appropriate for use in ileal Crohn's disease. We prefer a time dependent slow release oral 5-aminosalicylate drug for a more proximal distribution in ileal Crohn's disease instead of sulfasalazine. Eudragit coated preparations also have a suitable distribution of the drug for ileocolonic Crohn's disease. For patients with isolated colitis we favor sulfasalazine, especially for involvement of left colon and concomitant seronegative arthritis. 

Published evidence and clinical experience with antibiotics together suggest a modest benefit in Crohn's disease, particularly in colonic disease, but not for isolated small intestinal disease [15]. Antibiotics are not recommended in mild to moderate ileocaecal Crohn's disease for remission induction or maintenance and should not be used in the absence of infectious complications. 

**Figure 1:**
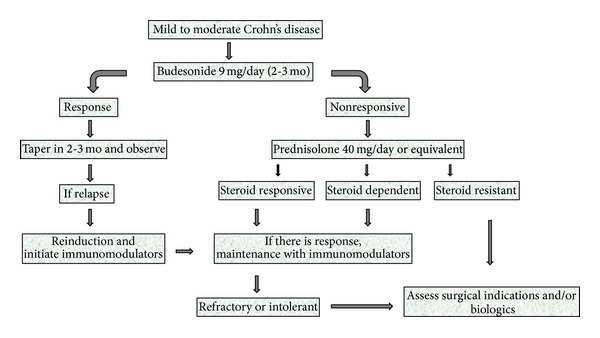
Algorithm for management of mild to moderate Crohn's disease.

In summary, remission induction can be achieved by budesonide or mesalamine in patients with mild to moderate disease (Figure 1). After remission continuing mesalamine or followup without a treatment are the options. If relapse occurs, patients can be treated by immunomodulatory therapy (azathioprine, 6-mercaptopurine, and methotrexate).

## 5. Moderate to Severe Ileocolonic Crohn's Disease

Moderate to severe disease activity is defined as mild to moderate Crohn's disease irresponsive to first line therapy, with presence of 2 or more of the following systemic symptoms as fever, weight loss, abdominal pain, nausea and vomiting, or symptoms of anemia. These patients are treated by systemic corticosteroids (prednisolone 40–60 mg/day, methyl prednisolone 32–48 mg/day) until symptomatic relief. Most patients may benefit from outpatient therapy, but in case of high fever or oral intolerance intravenous therapy may be required and hospitalization is necessary. Symptomatic improvement is usually achieved by corticosteroids in a week or two; after that 2-3 weeks of continuation is necessary before beginning taper. Earlier taper may increase the risk of relapse [1, 2]. In most patients high dose corticosteroids (methyl prednisolone 32–48 mg/day or equivalent) are begun to be gradually (4-5 mg every week) tapered after 3-4 weeks. 

Followup without treatment or maintenance with mesalamine formulations is not suitable for patients with severe activity. Thiopurine analogues (azathioprine 2–2.5 mg/kg/day or its active metabolite 6-mercaptopurine 1–1.5 mg/kg/day) are required for maintenance of remission, especially in patients with previous relapse, and should be introduced with the start of corticosteroid therapy [16]. Regular monitoring of toxicity is necessary after initiation of these drugs, particularly for bone marrow suppression and hepatotoxicity. A complete blood count and liver transaminases should be obtained frequently for the first month and then on a regular basis for as long as the patient is receiving therapy. Although not necessarily required, initial genotype testing for thiopurine methyltransferase is suggested by the United States Food and Drug Administration prior to use of thiopurines. If thiopurine methyltransferase genotyping is not available, treatment can be initiated with either drug at a dose of 50 mg/day and gradually increased, otherwise the drug can be started at a full dose. The dose of 6-mercaptopurine can be increased to a maximum of 1.5 mg/kg, and the dose of azathioprine can be increased to 2.5 mg/kg per day, provided there is no bone marrow suppression or hepatotoxicity. A response to these medications will usually be seen within three to six months; therefore, remission induction with thiopurines alone is not applicable.

Alternatively methotrexate (25 mg/week, intramuscular) may be chosen for maintenance of remission, if intolerance to thiopurine analogues occurs, but it should be kept in mind that methotrexate is contraindicated in pregnancy and in patients who have plans to conceive. Parenteral methotrexate is a valuable treatment choice in patients with steroid resistant or dependent patients with Crohn's disease [17], but there is no evidence on which to base a recommendation for use of lower dose oral methotrexate [18]. The benefit of methotrexate in Crohn's disease patients who have failed thiopurines, and vice versa, is less clear. It is effective in both induction and maintenance of remission. Although methotrexate therapy is generally considered safe and well tolerated, there are several adverse effects that should be taken into account which include nausea, abnormalities in liver enzymes, opportunistic infections, bone marrow suppression, and interstitial pneumonitis [19]. Long-term use of methotrexate in rheumatic diseases is associated with hepatic fibrosis which depends upon the specific disease being treated, the dose, and the duration of therapy, as well as comorbid conditions such as viral hepatitis, obesity, diabetes, and alcoholism; however, the risk of hepatotoxicity in inflammatory bowel disease is considered to be too low according to limited data [20].

Infliximab and other tumor necrotizing factor alpha (TNF*α*) antagonists (adalimumab and certolizumab pegol) are effective treatment options in moderately to severely active Crohn's disease in either remission induction or maintenance therapy [21]. In most patients disease activity can be controlled by first line therapies which include remission induction with corticosteroids and maintenance with immunomodulators. Therefore, anti-TNF*α* therapies should be reserved for patients who are refractory despite adequate dose and duration of corticosteroids and immunomodulators. Also in patients with the features of poor prognosis biologic agents may be used in first line as indicated in top-down strategy. Biologic agents are used in 2 major indications: luminal and fistulizing disease. Infliximab, adalimumab, and certolizumab pegol are approved for induction and maintenance of remission in both luminal and fistulizing Crohn's disease. Certolizumab pegol is approved for use in the United States and Switzerland and is therefore not widely available in Europe.

Infliximab is a chimeric IgG1 monoclonal antibody comprised of human and murine sequences with high affinity and specificity against TNF*α*. There are a lot of studies which also include single center experiences that demonstrate the efficacy of infliximab in luminal Crohn's disease both in trials and clinical settings. Infliximab was approved after the results of 2 randomized controlled trials involving moderately to severely active Crohn's disease patients who had inadequate response to standard treatments [22, 23]. ACCENT I trial especially underlined the efficacy of infliximab in maintenance of remission [23]. Infliximab is given as a 5 mg/kg intravenous infusion over a 2-hour period at baseline, 2nd, and 6th week for remission induction followed by 5 mg/kg every 8 weeks thereafter for maintenance. 

Adalimumab is a fully human recombinant IgG1 monoclonal antibody against TNF*α* which has been approved for rheumatoid arthritis, psoriatic arthritis, ankylosing spondylitis, and Crohn's disease. An important difference from infliximab is that adalimumab is administered by subcutaneous injection. There are 3 pivotal studies (CLASSIC-I, CHARM, and GAIN) that lead to the approval of adalimumab in remission induction, maintenance therapy, and treatment of patients who had lost response to or were intolerant of infliximab, respectively [24–26]. In CLASSIC-II, which is the follow-up study of CLASSIC-I, sustained remission after induction therapy was demonstrated [27]. Recommended induction dosing of adalimumab in Crohn's disease is 160 mg given subcutaneously initially at week zero and 80 mg at week two, followed by a maintenance dose of 40 mg every other week.

Certolizumab pegol (Cimzia) is a humanized monoclonal antibody Fab fragment linked to polyethylene glycol against TNF*α*. Polyethylene glycol increases its half-life and therefore reduces the need for frequent dosing. Unlike infliximab and adalimumab, certolizumab pegol does not have a Fc part that means a diminished activation of the complement pathway. The clinical significance of this feature is undetermined. The approval of certolizumab pegol in Crohn's disease is based on the results of 2 randomized controlled trials [28, 29]. It is effective in both remission induction and maintenance therapy of Crohn's disease and administered at a dose of 40 mg at zero, 2nd, and 4th weeks initially and subsequently on a monthly basis, subcutaneously. Whatever biologic agent is used, a search for active or latent tuberculosis is necessary to start timely prophylaxis if necessary.

Although biologic agents as a group clearly have favorable efficacy and safety profiles in clinical trials, clinical response and remission rates of 50–60% and 30–40%, respectively, are realistically achievable in patients with Crohn's disease. 

**Figure 2:**
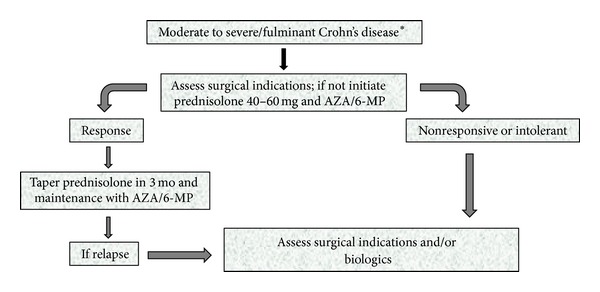
Algorithm for management of moderate to severe/fulminant Crohn's disease. *Top-down strategy with biologics may be more appropriate in selected patients with risk factors.

If medical therapies are demonstrated to be uneffective, surgical treatment options should be considered. Some authors suggest an indication for surgical resection initially in selected patients with short segment intestinal involvement, instead of a trial for biologic agents in immunomodulatory refractory Crohn's disease. This may be considered to be safe and cost-effective in such patients with localized ileocaecal disease, and threshold to decide for surgery may be kept low in this situation, especially if a patient has obstructive symptoms. As a first principle of management of Crohn's disease, medical and surgical treatment options should be discussed with a patient when the initial diagnosis is made and during the followup thereafter. Indication and timing of surgery is determined according to patient preferences and joint evaluation and decision of the gastroenterologist and surgeon. Management strategy of moderate to severe CD is summarized in Figure 2.

## 6. Severe-Fulminant Crohn's Disease

Severe-fulminant Crohn's disease is present if there is persisting symptoms despite conventional therapy such as high fever, severe nausea and vomiting, and abdominal pain or in the presence of intestinal obstruction, peritoneal irritation signs, and intraabdominal abscess. These patients unarguably should be hospitalized due to urgency and variety of their medical conditions and possible high risk of complications during admission or followup. In the presence of intestinal obstruction, intraabdominal abscess, or peritoneal irritation signs, abdominal imagings (plain abdominal radiograph, ultrasonography, and computerized tomography) and surgical consultation should be obtained promptly. An intraabdominal abscess requires drainage by surgery or radiologic intervention and intravenous antibiotics that cover gram-negative and anaerobic enteric microorganisms. After excluding intraabdominal abscess and fistula, parenteral corticosteroid (40–60 mg methyl prednisolone or equivalent) is started. If a patient has a resistant disease to corticosteroids or maintenance therapy by immunomodulators is not effective, biologic agents are the only medical treatment option.

## 7. Oral, Esophageal, and Gastroduodenal Involvement in Crohn's Disease

Gastroduodenal involvement is uncommon in patients with Crohn's disease; however, reported incidence in the literature is variable as 0.5% to 13% [30]. In a large series from Europe, it has been reported that 72 (7.7%) of 940 patients with Crohn's disease have proximal involvement [31]. Oral and esophageal involvement was far less common. In most of these patients there is accompanying distal intestinal and/or colonic involvement. Gastroduodenal Crohn's disease mostly affects distal antrum and duodenum, frequently as a *Helicobacter pylori* negative ulcer disease; therefore, it may easily be confused with peptic ulcer disease in which clinical presentation is also similar. Proton pump inhibitor therapy may improve symptoms, but remission can only be achieved by corticosteroid therapy. Mesalamine preparations and budesonide are not expected to be effective due to inadequate distribution in upper segments of the gastrointestinal tract. Most patients achieve remission by corticosteroid therapy; however, maintenance therapy with immunomodulators is frequently required. In a previous report, more than 90% of the patients with gastroduodenal Crohn's disease attained remission by medical treatment [32]. Anti-TNF*α* drugs are the best treatment of choice in cases refractory to standard therapies.

## 8. Colonic Involvement in Crohn's Disease

Mild isolated colonic Crohn's disease can be treated by sulfasalazine or other 5-aminosalicylate formulations; however, most patients with moderate to severe disease may need corticosteroid therapy for induction of remission. As mentioned before, 5-aminosalicylate use is not recommended by the ECCO guideline [2]. In the latest meta-analysis [13], it is emphasized that 5-aminosalicylates are a bit more effective than placebo for remission induction, but there is no role for maintenance therapy. 5-Aminosalicylates are widely used because of their relative safety compared to other drugs such as corticosteroids, immunomodulators, and biologic agents. We suggest using 5-aminosalicylates in patients with Crohn's colitis particularly in mild disease and because of the potential chemopreventive benefits against colon cancer; in fact this is also controversial [14]. Topical mesalamine can be considered for left-sided colitis as an adjunctive therapy but also this approach is not widely accepted due to inadequate efficacy data. Systemic corticosteroid therapy is very effective and therefore is the first line treatment in inducing remission, whereas budesonide has no role in treating colonic involvement due to proximal distribution of the drug. Immunomodulators especially azathioprine and 6-mercaptopurine are steroid-sparing agents for those who have a high risk of relapse. 

Antibiotics have always been one of the most frequently used drugs in management of inflammatory bowel diseases. Improving inflammatory response by reducing bacterial antigens in the colon has been the primary motive to use antibiotics in clinical trials. There are a lot of studies that evaluate the efficacy of different antibiotics in Crohn's disease, either alone or in combination. The most studied antibiotics are metronidazole, ciprofloxacin, rifaximin, and antimycobacterial drugs; therefore, it is difficult to establish whether any particular antibiotic is effective in Crohn's disease. A recent meta-analysis suggested a modest benefit, particularly in colonic disease [15]. A pooled analysis of antibiotic therapies by American Collage of Gastroenterology IBD Task Force recommended against its use in Crohn's disease, especially because of significant but limited efficacy and low quality of evidence [33]. In clinical practice, it is not realistic to expect achieving remission solely by antibiotic therapy, even in isolated colonic involvement. Aiming to symptomatic improvement is more practical. With the availability of more effective drugs to induce and maintain remission, and because of the risk of bacterial resistance, currently there is no place for antibiotics except for septic complications, symptoms attributable to bacterial overgrowth, or perianal disease. 

Anti-TNF*α* therapy or surgery should be discussed with patients if steroid resistance or failure in maintenance immunomodulatory therapy is in question. Occasionally colonic disease may be so severe that resection of the involved segment is necessary before any medical therapies can be used safely.

## 9. Perianal and Fistulating Disease

Perianal and/or fistulating disease is seen approximately in 1/3 of the patients with Crohn's disease. Perianal involvement can manifest as anal fissures, ulcers, perianal abscess, and fistulas. In these patients with anal fissure or ulcer, initial aim of treatment should be symptomatic improvement which includes sitz baths and preventing diarrhea to decrease local irritation. Anal region should be kept clean and dry after each bowel movement; bathing following defecation may be helpful. Patients should be treated by anti-inflammatory and immunomodulatory drugs for accompanying intestinal disease. 

Fistulating disease is one of the most serious challenges in management of Crohn's disease for both patients and physicians. Considerable amount of the patients with fistulating disease develop fistula before being diagnosed with Crohn's disease [1]. Fistulating disease is generally classified as perianal and nonperianal which includes fistulae communicating with other viscera (urinary bladder, vagina), loops of intestine (enteroenteral or enterocolonic fistulae), or the abdominal wall (enterocutaneous fistulae) [34]. This condition deteriorates patients' quality of life and is generally difficult to manage.

Presence of perianal symptoms should be questioned and assessed by imaging in every patient to detect perianal involvement before complications occur. Diagnostic assessment of fistulating perianal disease is essential for proper management of a patient. Examination under anaesthesia, fistulography, anorectal ultrasonography, pelvic computerized tomography, and magnetic resonance imaging (MRI) are the diagnostic tools that have been traditionally used. Rectosigmoidoscopy is also valuable in assessing degree of inflammation and detecting internal orifice of fistula in the rectum. Examination under anaesthesia is accepted to be the gold standard for evaluation of the anatomy of fistulas. It has the advantage of concomitant surgical intervention if complicated fistula or abscess is found. Current guidelines suggest pelvic MRI for the initial investigation of perianal disease, taking into account its accuracy and noninvasive nature, although it has been traditionally accepted to be inferior to examination under anaesthesia [34]. In fact both methods are not routinely recommended for a simple fistula, particularly if perianal abscess is not suspected. But whenever a patient has perianal pain, an abscess should always be excluded by imaging. Anorectal ultrasonography is a valuable tool in experienced hands. It has the advantage of being performed by a surgeon or gastroenterologist promptly as a part of physical examination. Any of these methods can be combined with the other to obtain more information. If an abscess is noticed by examination or imaging, surgical or radiological drainage is performed when necessary. A small abscess or fluid collection (with a diameter less than 1-2 cm) may not require drainage, and followup under antibiotics and immunomodulators can be sufficient.

Corticosteroids, the first line therapy in remission induction of Crohn's disease, are not recommended due to delaying fistula closure and increased requirement for surgical intervention [35]. Recommended first line medical therapy for fistulating disease is antibiotics. In fact, evidence for using antibiotics for fistulating disease mostly comes from uncontrolled case series and clinical experience. A response to treatment can be categorized as reduced drainage, cessation of drainage, or total fistula closure. It can be anticipated to have a response at about half of the patients treated by antibiotics, but generally complete healing should not be expected. 

Most studied antibiotics are metronidazole and ciprofloxacin, respectively. As it was shown in an open label case series by Bernstein et al., usually response with metronidazole (250–500 mg tid) can be achieved by 4–8 weeks; however, treatment is recommended to be continued for 3-4 months since relapse after cessation of therapy is universal [34–36]. Longer duration or maintenance of antibiotics may be needed in some cases. Another placebo controlled study of metronidazole investigated efficacy of topical metronidazole against placebo [37]. Patients treated with metronidazole ointment have reported significantly less perianal discharge but there was no improvement in the Perianal Crohn's Disease Activity Index (the primary endpoint), the patient's global impression of improvement, or quality of life scores. Ciprofloxacin (500 mg bid) was also shown effective alone or in combination with metronidazole in several studies, but randomized controlled trials are also lacking [38]. A recent randomized placebo controlled study by Thia and colleagues [39] investigated the efficacy of metronidazole and ciprofloxacin in patients with perianal fistulating Crohn's disease. Both antibiotics proved no significant benefit over placebo in either remission or response. The effects of combination therapy with ciprofloxacin and infliximab have also been assessed in a placebo controlled trial including 22 patients. In this study, combination of ciprofloxacin and infliximab tended to be more effective than infliximab alone but there was no significance (OR = 2.37, CI: 0.94–5.98, and *P* = 0.07) [40]. Even though evidence is lacking, clinical experience suggests that antibiotics are effective for improving symptoms, and exacerbation is so common that maintenance therapy has a critical importance. This issue was addressed in a study by Dejaco and colleagues [41]. Fifty-two patients with perianal fistulating Crohn's disease were treated by metronidazole or ciprofloxacin for 8 weeks. Patients receiving azathioprine concomitantly achieved a better response at week 20 than those who do not. 

We agree with the current guidelines [33, 34] and prefer to initiate antibiotic therapy (metronidazole and/or ciprofloxacin) with azathioprine (2–2.5 mg/kg/day) simultaneously. After 3–6 months of combination therapy we try to stop antibiotics. If a relapse occurs under maintenance thiopurine therapy, biologic agents are indicated. Although infliximab, adalimumab, and certolizumab pegol are all approved for treatment of luminal Crohn's disease, only infliximab and adalimumab have been proved to be effective in perianal fistulating disease. Infliximab was the first agent proved to be effective for inducing fistula closure and for maintaining this response in a randomized controlled trial [42]. The results of the ACCENT II trial also have confirmed these findings by demonstrating 69% response rate at week 14 and 36% remission rate at week 54 [43]. 

In the CHARM trial which evaluates the efficacy of adalimumab in maintenance therapy of Crohn's disease, 117 patients had active perianal fistulae [25]. All patients received 80/40 mg adalimumab at weeks 0 and 2, and at week 4 they were randomized to receive adalimumab either 40 mg weekly, 40 mg every other week, or placebo for a year. Fistula remission rates in patients receiving adalimumab were significantly better than those in placebo group at week 26 and 56 (30% versus 13% and 33% versus 13%, resp.). The efficacy of adalimumab in perianal Crohn's disease was recently shown in an open label study by Fortea-Ormaechea and colleagues [44]. At the end of followup, the percentage of patients with partial and complete response was reported to be 35% and 41%.

In PRECISE 1 and PRECISE 2 trials that investigate the efficacy of certolizumab pegol in Crohn's disease, subgroup analysis of patients with draining fistula showed similar rates of remission in treatment and placebo arms, although the studies were not powered to show a difference in a secondary endpoint [28, 29]. Currently, there is no randomized controlled data to recommend certolizumab pegol in treatment of patients with perianal fistulating disease. 

**Figure 3:**
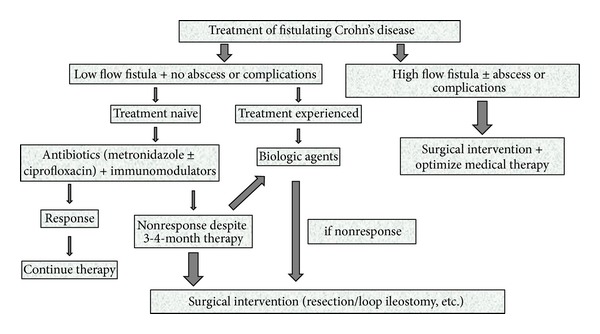
Algorithm for management of fistulating Crohn's disease.

Current guidelines recommend multidisciplinary approach and patient followup together by a gastroenterologist and a surgeon, especially for patients with fistulating Crohn's disease. Surgical treatment is seldom needed for simple fistulae, but is always necessary for a complex perianal disease. Most performed surgical interventions include abscess drainage and seton placement. Seton placement is very effective in improving symptoms by maintaining patency of tract and preventing abscess recurrence. Combination of infliximab and seton placement seems to be better than either method alone, but excluding perianal abscess before initiating anti-TNF*α* therapy is very important to avoid septic complications [45, 46]. Instead of cutting setons, loose setons should be used in patients with Crohn's disease. Fistulectomy and fistulotomy are not performed in routine practice, because of the risk of incontinence. In a group of patients with high flow fistulae, especially in enterocutaneous, enterovesical, or enterovaginal fistulae, medical management may not be successful. There is a notable lack of controlled data for those with nonperianal fistulae. In these patients, a diverting stoma or resection of the fistula tract and diseased intestinal segment may be necessary. Determination of disease activity and optimizing medical therapy should always accompany surgical intervention. Management of fistulating Crohn's disease is summarized in Figure 3.

## 10. Management of Stricturing Crohn's Disease

In Crohn's disease, development of strictures in gastrointestinal tract is a common problem which may lead to severe complications and frequent hospitalizations. Patients may present either with mild, intermittent symptoms as abdominal pain and nausea or severe ileus and acute abdomen. Plain abdominal radiography may give clues regarding the cause for symptoms and if they are attributable to obstruction. Presence of air in small bowel, multiple air-fluid levels, and distended intestinal loops are important clues for obstruction even in mild symptomatic or asymptomatic patients. Free air under diaphragm means perforation and requires prompt surgical intervention. In patients with mild symptoms, detecting the level of the obstruction by imaging modalities such as small bowel series and computerized tomography (CT) with oral and intravenous contrast agent is necessary. As an alternative, enteroclysis is a double-contrast radiographic study that is performed by passing a tube into the proximal small bowel and injecting both barium and methylcellulose. Although it provides high quality images that are superior to small bowel series, enteroclysis is an expensive, time- and personnel-demanding method. CT and MR enterography are novel and most promising techniques to visualize both intestinal lumen and walls. Nasogastric decompression, intravenous fluid and electrolyte replacement, parenteral nutrition, and antibiotics—if signs of infection are observed—are essential for patients with ileus. An improvement can be achieved by this approach in a considerable proportion of patients in 24–48 hours. Immediately after relief of ileus an abdominal CT should be obtained to disclose level of obstruction or possible intraabdominal complication.

Some patients have an inflammatory stenosis as cause of obstruction which may respond to intravenous corticosteroids or biologics. Therefore it is important to determine nature of obstruction by a gadolinium enhanced MRI which can differentiate inflammatory or fibrotic strictures. Management strategy is organized according to nature (inflammatory or fibrotic), localization, and length of stricture, presence of abscess or fistula, and suspicion of concomitant malignancy especially in colonic involvement. Experience of an institution is another factor that influences management strategy. Medical treatment must be optimized in case of increased CRP and/or endoscopically persistent mucosal disease, otherwise surgery is the only option for a fixed and fibrotic stricture. Endoscopic balloon dilation is an appropriate technique for the management of accessible short strictures that are less than 5 cm long; however, perforation risk is high and 24 h surgical service is essential. In a recent review including 347 patients from 13 studies by Hassan and colleagues [47], technical success rate was reported to be 86% and long-term clinical efficacy was 56%. Stricturoplasty is a suitable method for intestinal strictures which are less than 10 cm long. If a longer intestinal segment is involved or there is a concomitant abscess or fistula, partial resection is preferred. Endoscopic balloon dilation and stricturoplasty are not recommended for colonic strictures due to high risk of perforation and risk of malignancy on the long term.

## 11. Management of Pregnant Patients

It is recognized that pregnancy does not influence the course of inflammatory bowel disease. The course of Crohn's disease during pregnancy appears to be determined in part by the activity of the disease at conception. It can be anticipated that patients in clinical remission before pregnancy are very much likely to remain in remission also during pregnancy [48]. There is an increased risk for low birth weight infants and premature delivery in patients with active disease. Therefore patients should be informed to have a planned conception during clinical remission. Generally, it is considered to be safe to perform flexible sigmoidoscopy during pregnancy [49]. Colonoscopy also seems to be safe, but due to limited experience it should be performed only when it is necessary. X-ray is contraindicated in pregnancy, unless it is essentially needed for diagnosis of a life-threatening condition. 

Use of 5-aminosalicylates, sulfasalazine, and corticosteroids is generally safe in pregnancy. Both 6-mercaptopurine and azathioprine cross the placenta and can be detected in cord blood. The largest experience with these drugs in pregnancy has been derived from patients with solid organ transplantation. Although there are reports of teratogenic effects on fetus, it is important to continue therapy as the benefits of controlled disease far outweigh the risk of teratogenicity. Methotrexate is contraindicated in pregnancy, and uninterrupted contraception is necessary during its use. Both male and female patients using methotrexate should discontinue this drug and use contraception for at least three months prior to conception.

Some experts used to suggest that male patients stop immunosuppressive therapy 3 months before planned conception, but this recommendation was based on the findings of a previous retrospective study [50]. A more recent report suggested that no interruption for thiopurines is needed for male patients before conception [51].

There is insufficient data about the use of biologics in pregnancy. Infliximab and adalimumab can cross the placenta; however, limited evidence suggests that there is no risk for these drugs. There is no data published on the use of certolizumab pegol in pregnancy. Currently, anti-TNF*α* drugs should only be used in pregnancy when it is clearly needed and if there is no alternative. If it is decided to use biologics during pregnancy, treatment may best be interrupted during the third trimester in order to prevent circulating anti-TNF antibodies in the newborn.

## 12. Preventing Postoperative Recurrence in Crohn's Disease

There is still some controversy regarding the prevention of recurrence after surgery in Crohn's disease. However, there is a consensus about the need for prophylactic therapy to prevent recurrence. Prophylactic therapy may not be necessary only for a minority of patients. For most patients, particularly when a risk factor of recurrence is present, prophylactic therapy is required. These risk factors include presentation at a young age, smoking, fistulating/perianal disease, extensive small bowel disease, and requirement for early surgery. It is strongly recommended to evaluate disease activity by ileocolonoscopy within the first year of surgery whatever prophylaxis is started. Mesalamine preparations can be used for prophylaxis in patients with colonic involvement, but its efficacy is similar to placebo. Metronidazole is significantly effective for the prevention of postoperative recurrence, but its use is limited by side effects. In clinical practice, metronidazole is initiated at a dose of 750–1500 mg/day immediately after surgery and continued for 3 months or as tolerated. Thiopurines are the cornerstone of the prophylactic treatment of postoperative recurrence. Prophylaxis with biologic agents can be considered for patients who require surgery under thiopurine therapy. However, this approach has not been validated in randomized controlled trials. If the diseased segment is fully resected, continuing therapy with thiopurines seems safer and more practical. 

## 13. Failure of Anti-TNF Therapy

There are two kinds of treatment failure in patients receiving biologic agents. Primary failure describes a lack of response in biologic naïve patients. It is typically accepted to wait until the end of induction phase of therapy to assess degree of response [52]. The last ECCO guideline however suggests determining primary lack of response within 12 weeks [2]. Secondary failure is defined as a loss of response in patients who previously improved with anti-TNF therapy. There is a lack of evidence for switching between biologic agents if primary lack of response is in question, but in most situations there are not much alternatives. It is important to assess the need for surgery in these patients before switching therapy. Secondary loss of response mostly appears due to formation of antibodies against anti-TNF molecules; therefore, dose escalating or reduction in interval between doses may be effective [2]. Switching is an effective alternative but reduces future therapeutic options.

## 14. Combination of Anti-TNF and Immunomodulatory Therapies

Primary intention behind the approach of combination of immunomodulatory drugs with biologic agents is based on expectance of a better clinical response and less antibody formation against infliximab [53]. However, combination therapy brings an increased risk of life-threatening infections and malignancy. The best data favoring combination treatment comes from the SONIC study in which infliximab and azathioprine combination was demonstrated to be more effective in inducing clinical and endoscopic remission than infliximab and azathioprine monotherapies [7]. Another study by Van Assche and collegues [54] evaluated the efficacy of combination therapy from a different perspective. It was shown that discontinuation of immunosuppressive drug (azathioprine/6-mercaptopurine or methotrexate) after 6 months of combination therapy did not affect clinical response or remission rates within 2 years of followup. These data suggest that combination therapy is the best option for remission induction, but after 6–12 months of treatment, discontinuation of either immunomodulatory or biologic agent can be considered according to patient characteristics as being immunomodulatory naïve or not. 

Management of Crohn's disease requires a multidisciplinary approach including a gastroenterologist, surgeon, and radiologist. After initial diagnosis it is crucial to inform patients about the course, prognosis, complications, and treatment options of Crohn's disease; patients should be encouraged to participate in decision-making for the management of this life-long disease.

## Figures and Tables

**Table 1:** A simplified classification of severity in Crohn's disease.

Severity of symptoms	CDAI	Description
Clinical remission	<150	Spontaneous or posttreatment remission
Mild to moderate Crohn's disease	150–220	Good oral intake, mild symptoms, and absence of dehydration. Ambulatory followup is sufficient.
Moderate to severe Crohn's disease	220–450	Irresponsiveness to first line therapy, presence of systemic symptoms.
Severe-fulminant Crohn's disease	>450	Persisting symptoms despite therapy or presence of high fever, obstruction symptoms, peritoneal signs, cachexia, or intraabdominal abscess.

**Table 2:** Characteristics of the drugs used in treatment of Crohn's disease.

Drugs	Utility	Severity of disease	Disease localization	Recommendation [33]	Dose	Duration
Mesalamine	RI	Mild	Distal ileum, colon	2C	3-4 g/day	NA
Budesonide	RI	Mild to moderate	Distal ileum, caecum	1C	9 mg/day	3–6 months
Systemic corticosteroids	RI	All	All	1C	40–60 mg/day*	3-4 months
Antibiotics	RI/M	Perianal/fistulating	Ileocolonic	2C	NA	3–6 months
Thiopurines						
Azathioprine	M	All	All	2C	2–2.5 mg/kg	Indefinite
6-MP					1–1.5 mg/kg	
Methotrexate (i.m.)	RI/M	All	All	2C	25 mg/week	Indefinite
Biologics						
Infliximab	RI/M	Moderate to severe/fistulating	All	1B/C	5 mg/kg/dose	Indefinite
Adalimumab					40 mg/dose	
C. pegol					400 mg/dose	

RI: remission induction; M: maintenance; *prednisolone or equivalent; NA: not applicable; A: high-quality evidence; B: moderate-quality evidence; C: low-quality evidence; D: very-low-quality evidence; 1: strong recommendation; 2: week recommendation.
